# Xyloglucan Fucosylation Modulates *Arabidopsis* Cell Wall Hemicellulose Aluminium binding Capacity

**DOI:** 10.1038/s41598-017-18711-1

**Published:** 2018-01-11

**Authors:** Jiang-Xue Wan, Xiao-Fang Zhu, Yu-Qi Wang, Lin-Yu Liu, Bao-Cai Zhang, Gui-Xin Li, Yi-Hua Zhou, Shao-Jian Zheng

**Affiliations:** 1 State Key Laboratory of Plant Physiology and Biochemistry, College of Life Sciences, Zhejiang University, Hangzhou, 310058 China; 2 State Key Laboratory of Soil and Sustainable Agriculture, Institute of Soil Science, Chinese Academy of Sciences, Nanjing, 210008 China; 3 State Key Laboratory of Plant Genomics, Institute of Genetics and Developmental Biology, Chinese Academy of Sciences, Beijing, 100101 China; 4 College of Agronomy and Biotechnology, Zhejiang University, Hangzhou, 310058 China

## Abstract

Although xyloglucan (XyG) is reported to bind Aluminium (Al), the influence of XyG fucosylation on the cell wall Al binding capacity and plant Al stress responses is unclear. We show that *Arabidopsis* T-DNA insertion mutants with reduced *AXY3* (*XYLOSIDASE1*) function and consequent reduced levels of fucosylated XyG are more sensitive to Al than wild-type Col-0 (WT). In contrast, T-DNA insertion mutants with reduced *AXY8* (*FUC95A*) function and consequent increased levels of fucosylated XyG are more Al resistant. *AXY3* transcript levels are strongly down regulated in response to 30 min Al treatment, whilst *AXY8* transcript levels also repressed until 6 h following treatment onset. Mutants lacking *AXY3* or *AXY8* function exhibit opposing effects on Al contents of root cell wall and cell wall hemicellulose components. However, there was no difference in the amount of Al retained in the pectin components between mutants and WT. Finally, whilst the total sugar content of the hemicellulose fraction did not change, the altered hemicellulose Al content of the mutants is shown to be a likely consequence of their different XyG fucosylation levels. We conclude that variation in XyG fucosylation levels influences the Al sensitivity of *Arabidopsis* by affecting the Al-binding capacity of hemicellulose.

## Introduction

Aluminium (Al) toxicity is the major constraint for crop production in acid soils^1^. When the soil pH drops to below 5, Al becomes solubilized into soil solution and is absorbed by plant roots^2^. Al then interferes with a wide range of plant physical and cellular processes. For example, Al interacts with multiple root cell processes^3,4^. Al affects signal transduction pathways such as the plasma membrane phosphoinositide pathway^5^, thus disrupting cytosolic Ca^2+^ homeostasis and distorting cytoskeletal dynamics^6^, and finally resulting in functional and structural damage^3^. Although highly damaging, the exact mechanisms of Al toxicity remain poorly understood.

Nevertheless, to cause damage to plant roots, Al must first enter the cellular cytosol. However, most of the plant Al content is bound to the cell wall^7,8^. For example, almost 90% of the total cultured tobacco cell Al is associated with the cell wall^9^, whilst 85–90% of total accumulated barley root Al is also tightly bound to the cell wall^10^. The pectin component of the plant cell wall was long considered to be a likely major cell wall Al binding site because its negatively charged carboxylic groups have high affinity for Al^3+^ ^9,11^. However, recent studies have shown that hemicellulose is not only susceptible to Al stress in wheat^12^, triticale^13^ and rice^14^, but also acts as the principal Al binding site. Furthermore, the Arabidopsis xyloglucan hemicellulose component has recently been shown to be a much more effective binder of Al than pectin^15,16^, although the exact mechanism of how xyloglucan can bind Al is has remained unclear. Thus, the role of xyloglucan (XyG) in Al toxicity/tolerance and the underlying physiological and molecular mechanisms require further investigation.

XyG is the major hemicellulosic polysaccharide in the primary plant cell walls of dicots and of non-gramineous monocots^17^, and to a lesser extent in grasses^18^. XyG consists of a β-1,4 linked glucan chain decorated with various heterogeneous side chains depending on plant species and tissue type^19^, and also frequently bears side-chains at the *O*-6 position^20^. The pattern of XyG substitutions at each backbone glucosyl residue is denoted using a single letter nomenclature^21^. For example, the letter G denotes an unsubstituted backbone β-D-Glcp residue, whilst X denotes a backbone Glc unit substituted with a xylosyl-residue [i.e., an α-D-Xylp-(1−6)- β-D-Glcp moiety]^19^. The X groups can carry further additional glycosyl-moiety decorations, and so far 17 different side chain structures have been identified^22^. In Arabidopsis, X groups can be decorated by a β-D-Galp residue (L side chain), which is often further decorated with an α-L-Fucp residue (F side chain) and/or an *O*-acetyl-substituent^19,23^. Thus, through the action of a XyG specific hydrolase (XEG)^24^, XyG can be released from the cell wall, thus giving semi-quantitative insights into the relative distributions of XyG side chains such as XXG, XLG, XFG, XXLG, XXFG, XLLG, XXXG and XLFG, based on oligosaccharide mass profiling (OLIMP)^17,25^. In addition to XyG, endotransglycosylases (XETs), which are involved in the remodeling of XyG in the wall or the incorporation of newly synthesized XyG through cutting and religation of the XyG polymers^26^, and other proteins, such as expansins, are also known to cause cell wall creep^27^, thus contributing to plant growth^23^. Moreover, XyG oligosaccharides (oligos) themselves have also been shown to be part of this coordinated cell wall expansion^28^. For instance, the apoplastic glycoside hydrolase encoded by the *XYLOSIDASE1* (*XYL1* or *AXY3*) gene releases xylosyl residues from xyloglucan oligosaccharides at the non-reducing end. As a result, *axy3* mutants, having reduced apoplastic glycoside hydrolase activity, show reduced XyG fucosylation^29^. In contrast, the *AXY8* gene (previously designated as *FUC95A*; www.cazy.org) encodes a fucosidase belonging to the glycosylhydrolase family 95. As a result, *axy8* mutants, having reduced fucosidase function, exhibit increased XyG fucosylation^19^ (http://paulylab.berkeley.edu/axy-mutants.html). These changes in XyG fucosylation (in either *axy3* or *axy8* mutants) confer no change in visible growth or morphological phenotypes^19^. Whilst both XET and expansins have previously been demonstrated to be involved in plant responses to Al stress^15,30^, the possibility that modification of XyG structure also alters plant Al stress responses remains to be investigated.

Here we first show that *Arabidopsis* mutants with reduced *AXY3* or *AXY8* function have altered Al stress responses. Two T-DNA insertional mutants with reduced *AXY3* (*XYLOSIDASE1*) function and consequent reduced levels of fucosylated XyG displayed increased Al sensitivity. In contrast, two T-DNA insertional mutants with reduced *AXY8* function and consequent increased levels of fucosylated XyG displayed increased Al resistance. We next characterize the responses of *AXY3* and *AXY8* to Al stress, and show that modulation of the XyG fucosylation level by AXY3 and AXY8 changes the Al binding capacity of hemicellulose, which is the major contributor to Al retention in the *Arabidopsis* cell wall.

## Results

### Al stress changes XyG structure

**Figure 1:**
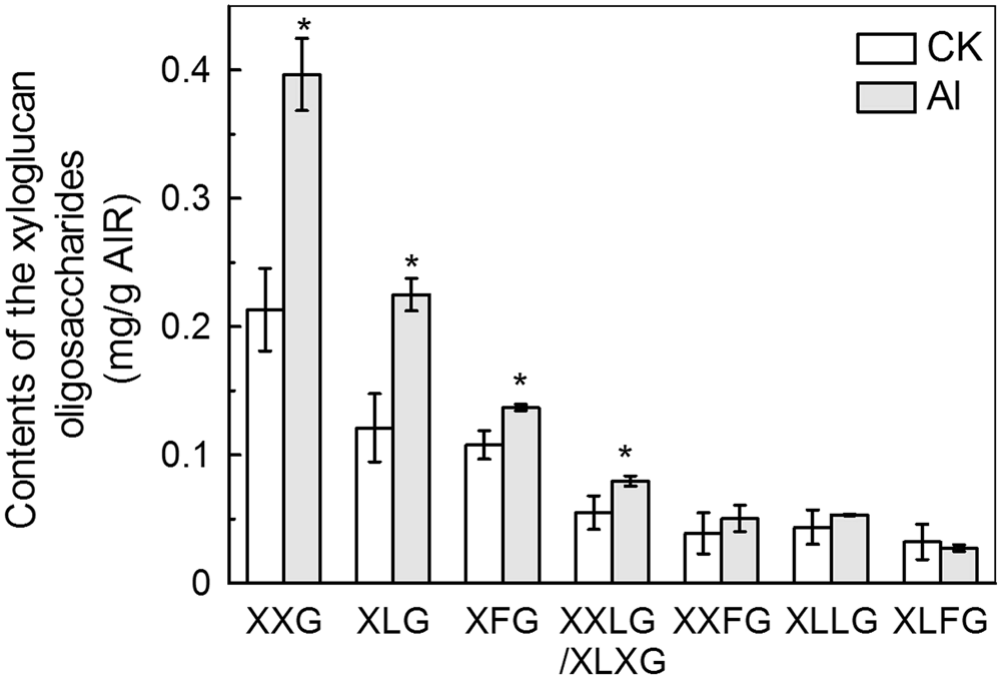
MALDI–TOF MS analysis of the relative abundance of xyloglucan oligosaccharides released by xyloglucanase. Cell wall materials were extracted from Al-untreated and Al-treated WT (Col-0) roots and digested with xyloglucanase. The oligosaccharides obtained were analyzed by MALDI–TOF MS. Data are means ± SD. n = 2.

Because XyG is a major component of cell wall hemicellulose and also the major Al binding site of the *Arabidopsis* cell wall^16^, we determined the effect of Al treatment on root cell wall XyG content. MALDI-TOF analysis after xyloglucanase digestion indicated that some xyloglucanase-accessible XyG repeat-units, especially XXG, XLG, XFG and XXLG (and/or XLXG), were increased significantly following growth for 24 hours in Al stress conditions (Fig. 1), meaning that there are more side chains in XyG when subjected to Al stress.

### Mutants with altered XyG structure differ in their Al sensitivities

**Figure 2:**
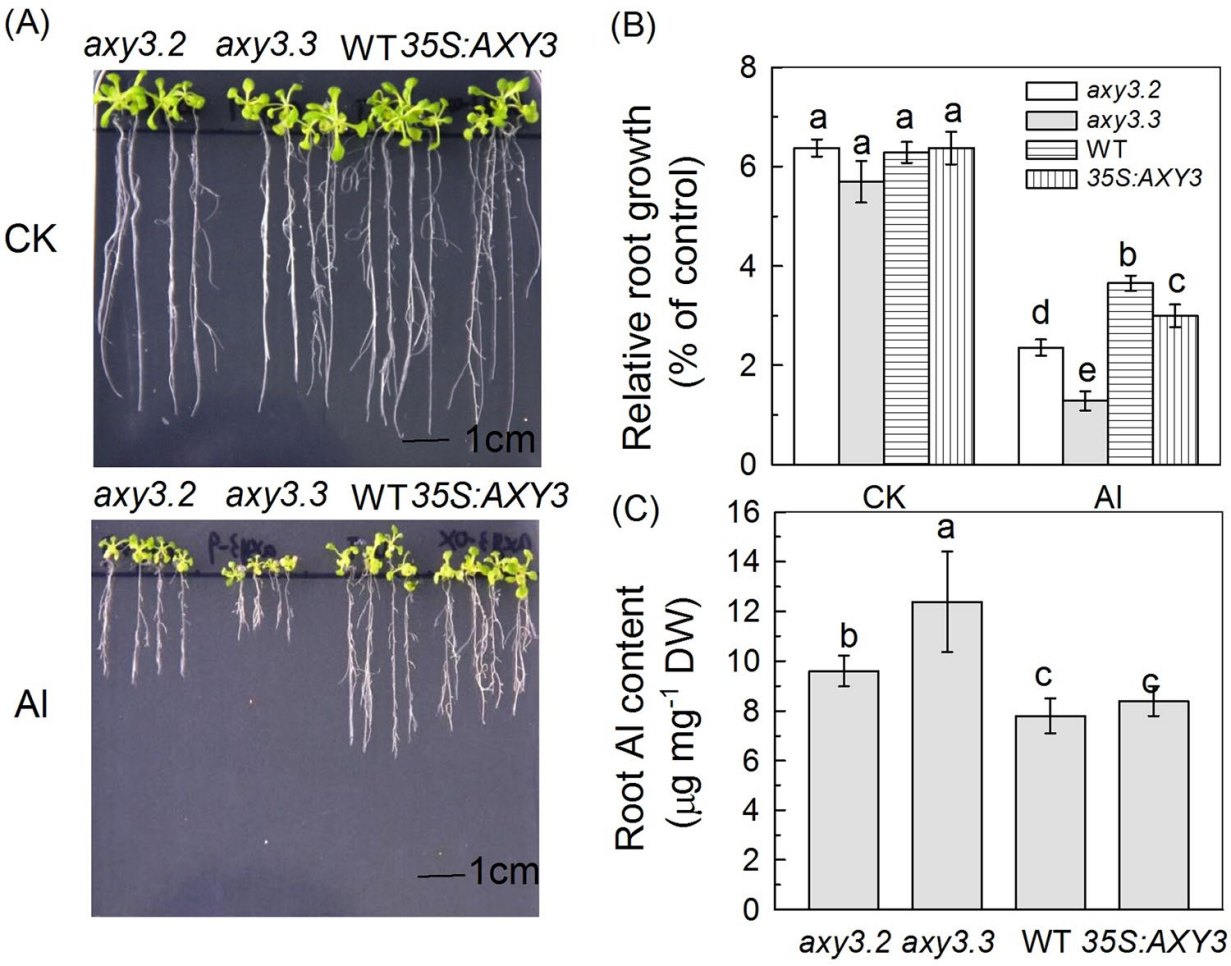
Phenotypes of the WT, *axy3* mutants, and lines overexpressing *AXY3*. (**A**) WT, *axy3* mutants and *AXY3* overexpression lines (*35 S:AXY3*) were grown on 1/2 MS plates in the presence or absence of 50 µM Al^3+^. Seedlings were treated when roots were about 1 cm long, immediately after germination. (**B**) Root elongation of WT, *axy3* mutants and *AXY3* overexpression lines in the presence or absence of 50 µM Al^3+^. (**C**) Root Al content of WT, *axy3* mutants and *AXY3* overexpression lines. Data are means ± SD (n = 4). Different letters show significant differences at P < 0.05 by Student’s *t* test.

**Figure 3:**
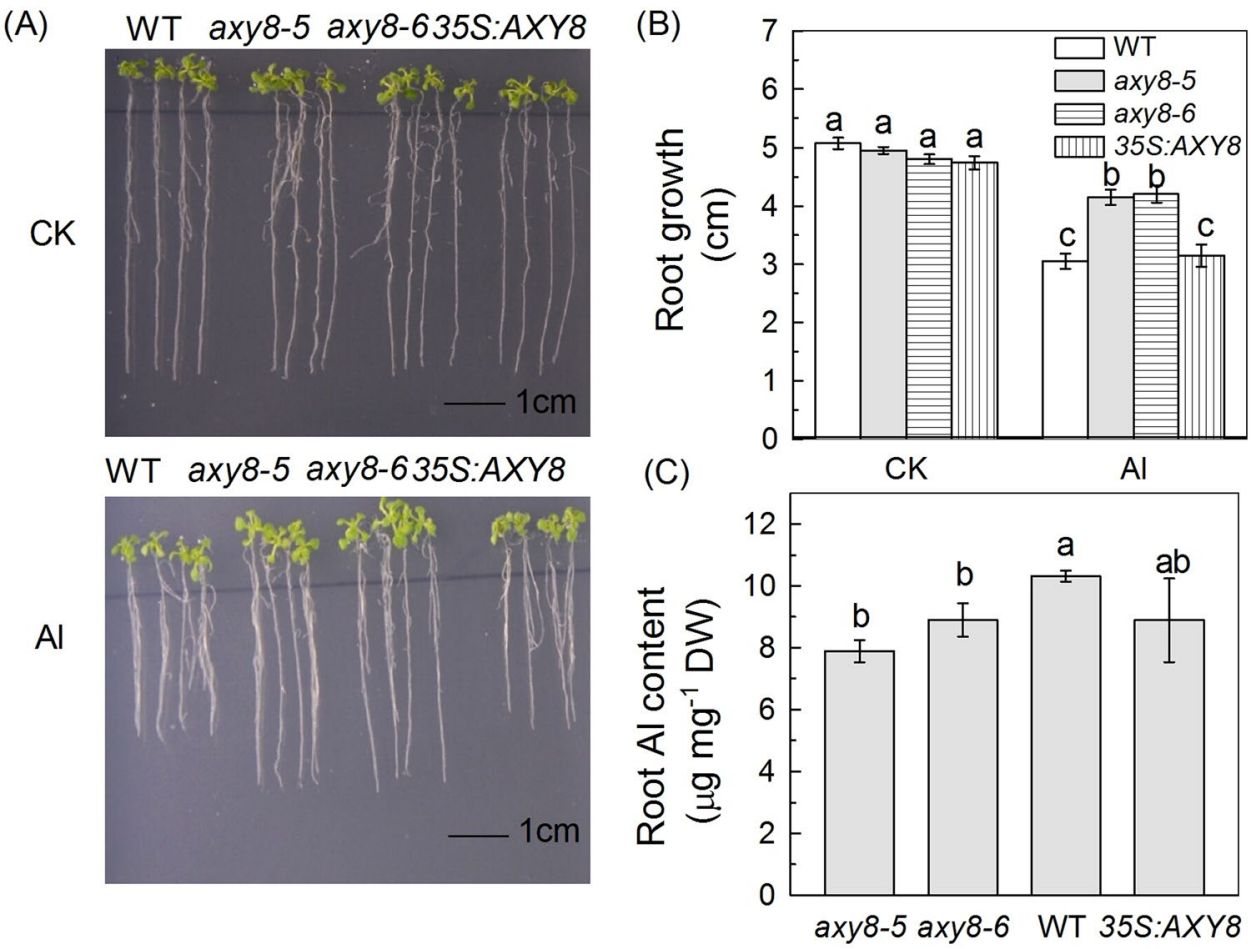
Phenotypes of the WT, *axy8* mutants, and lines overexpressing *AXY8*. (**A**) WT, *axy8* mutants and *AXY8* overexpression lines (*35 S:AXY8*) were grown on 1/2 MS plates in the presence or absence of 50 µM Al^3+^. Seedlings were treated when roots were about 1 cm long, immediately after germination. (**B**) Root elongation of WT, *axy8* mutants and *AXY8* overexpression lines in the presence or absence of 50 µM Al^3+^. (**C**) Root Al content of WT, *axy8* mutants and *AXY8* overexpression lines. Data are means ± SD (n = 4). Different letters show significant differences at P < 0.05 by Student’s *t* test.

To determine if XyG structure affects plant Al stress responses, we next tested the Al sensitivity of *Arabidopsis* T-DNA insertional mutants having altered *AXY3* or *AXY8* function and resultant altered XyG fucosylation levels. The mutants we tested were: *axy3.2* (GABI_749G08) and *axy3.3* (SAIL_916H10), both having reduced fucosylation; and *axy8-6* (GABI_440B01) and *axy8-5* (GABI_863G09), both having increased fucosylation. In addition, *AXY3* and *AXY8* overexpression lines (*35 S:AXY3* and *35 S:AXY8*) were also tested. Quantitative RT-PCR was first performed to confirm the expected alterations in *AXY3* and *AXY8* transcript levels in these various mutants and overexpression lines (Supplemental Fig. 1). We next found that whilst growth on agar medium containing 50 μM AlCl_3_ for 7 days inhibited wild-type (WT) Col-0 root growth by 34%, root growth of *axy3.2* and *axy3.3* was inhibited by 59% and 47% respectively (Fig. 2A and B). These observations indicate that reduced fucosylation renders these mutants more Al sensitive. In contrast, the *axy8-5* and *axy8-6* mutants were more Al resistant in these same conditions (Fig. 3A and B). Because Al sensitivity generally correlates with root Al content, we next measured the mutant root Al contents, and found that whilst the *axy3* mutants accumulated significantly more Al than WT (Fig. 2C), the *axy8* mutants accumulated less (Fig. 3C). Despite producing the expect increased levels of transcripts (Supplemental Fig. 1), the *AXY3* and *AXY8* overexpression lines did not display phenotypes that were the opposite to those displayed by their respective reduced function mutants (Figs 2 and 3). This latter observation suggests that the levels of the AXY3 and AXY8 proteins in WT might be sufficient to produce suitable amount of fucosylated XyG oligos for normal cell wall synthesis. Therefore, in the following experiments, we excluded the overexpression lines.

### Dose and Time–Response of AXY3 and AXY8 Expression to Al

**Figure 4:**
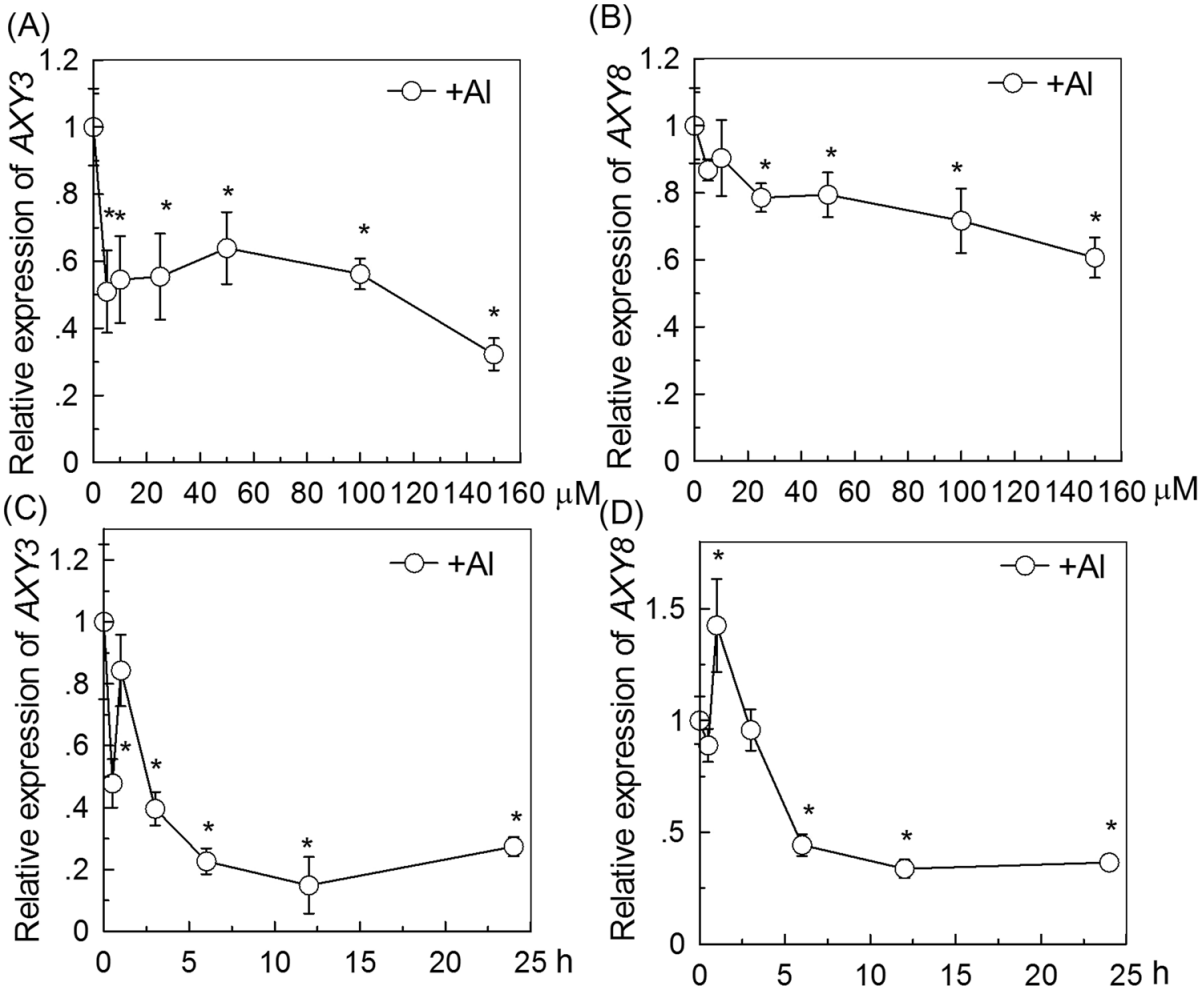
Al dose and time–responsive relative expression of *AXY3* and *AXY8* as measured by Quantitative RT-PCR. (**A**) *AXY3* expression in roots exposed to 0–150 µM Al^3+^ for 24 h. The Y axis shows *AXY3* RNA levels normalized to that of the control (0 µM Al^3+^). (**B**) *AXY8* expression in roots exposed to 0–150 µM Al^3+^ for 24 h. The Y axis shows *AXY8* RNA levels normalized to that of the control (0 µM Al^3+^). (**C**) *AXY3* expression in roots exposed to 50 µM Al^3+^ for 0-24 h. The Y axis shows *AXY3* RNA levels normalized to that of the control (50 µM Al^3+^ for 0 h). (**D**) *AXY8* expression in roots exposed to 50 µM Al^3+^ for 0–24 h. The Y axis shows *AXY8* RNA levels normalized to that of the control (50 µM Al^3+^ for 0 h). Values are mean ± SD (n = 3). The asterisks show significant differences between control and Al treatments at P < 0.05 by Student’s *t* test.

To next examine whether Al stress affects the levels of *AXY3* or *AXY8* transcripts, dose-response and time course experiments were performed. RT-qPCR analysis revealed *AXY3* expression (in WT roots) to be substantially repressed by an Al concentration as low as 5 µM (Fig. 4A), and by 50 µM Al within as short a length of time as 30 min exposure (although there was also a transient up-regulation at 1 h after exposure; Fig. 4C), suggesting that *AXY3* expression is very sensitive to Al stress. In contrast, the expression of *AXY8* was much less sensitive to Al, although repressed by 25 µM Al (Fig. 4B) and by 50 µM Al for 6 h (Fig. 4D).

### The *axy8* and *axy3* mutants have opposite root cell wall and hemicellulose Al contents

**Figure 5:**
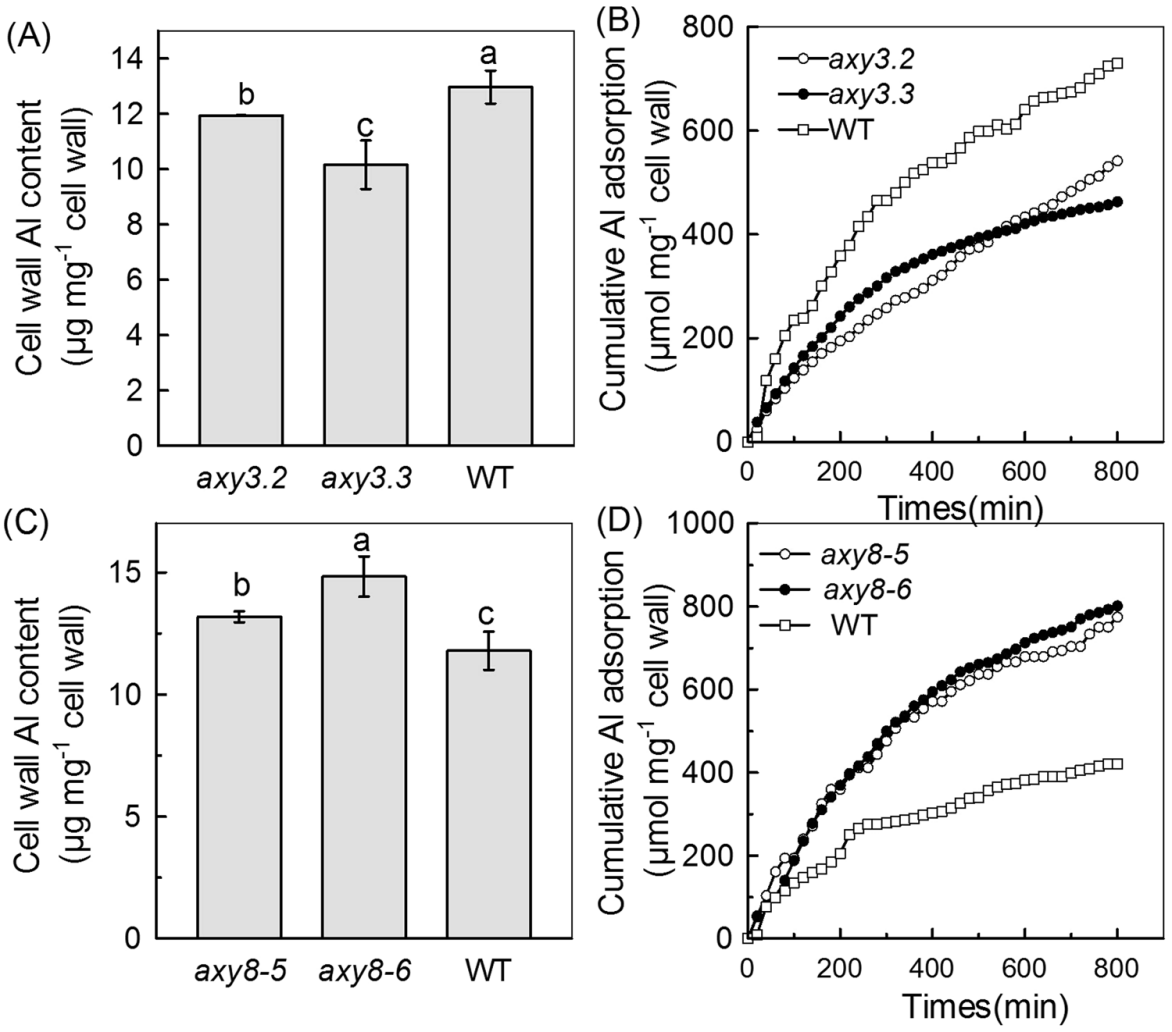
Cell wall Al content of the WT, *axy3* and *axy8* mutants. (**A**) Cell wall Al content of WT and *axy3* mutants in the presence of 50 µM Al^3+^ for 24 h. Data are means ± SD (n = 4). Different letters show significant differences at P < 0.05 by Student’s *t* test. (**B**) Cell wall adsorption kinetics of WT and *axy3* mutants in the absence of 50 µM Al^3+^. (**C**) Cell wall Al content of WT and *axy8* mutants. Data are means ± SD (n = 4). Different letters show significant differences at P < 0.05 by Student’s *t* test. (**D**) Cell wall adsorption kinetics of WT and *axy8* mutants in the absence of 50 µM Al^3+^.

Since the total root Al content of the *axy3* and *axy8* mutants differed from that of WT, we next determined the specific Al content and Al adsorption of root cell walls, and found that these were less than WT in *axy3* mutants but greater than WT in *axy8* mutants (Fig. 5). These results indicate that the varied fucosylation levels in WT versus mutants affects the Al binding capacity of the cell wall.

**Figure 6:**
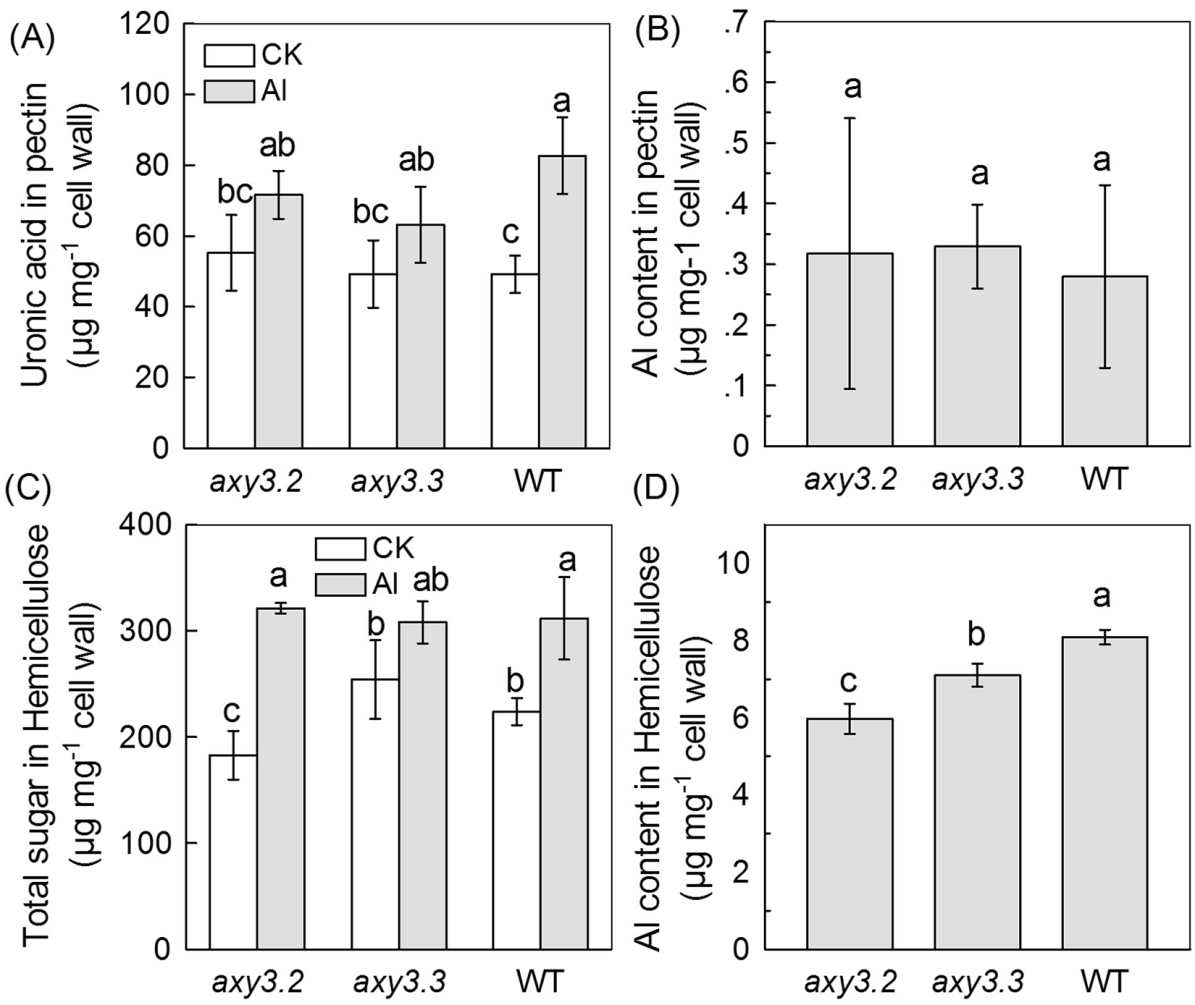
Pectin and hemicellulose uronic acid, total sugar and Al contentin WT and *axy3* mutants. (**A**) Uronic acid content in the extractable pectin of WT, *axy3.2* and *axy3.3* roots. Seedlings were treated with or without Al for 24 h. (**B**) Al content in the extractable pectin of WT, *axy3.2* and *axy3.3* roots. Cell wall materials from Al treated roots were fractionated into pectin (see Methods for details). (**C**) Total sugar content in the extractable hemicellulose of WT, *axy3.2* and *axy3.3* roots. Seedlings were treated with or without Al for 24 h. (**D**) Al content in the extractable hemicellulose of WT, *axy3.2* and *axy3.3* roots. Cell wall materials from Al treated roots were fractionated into hemicelluloses (see Methods for details). Data are means ± SD. n = 4. Different letters show significant differences at P < 0.05 by Student’s *t* test.

**Figure 7:**
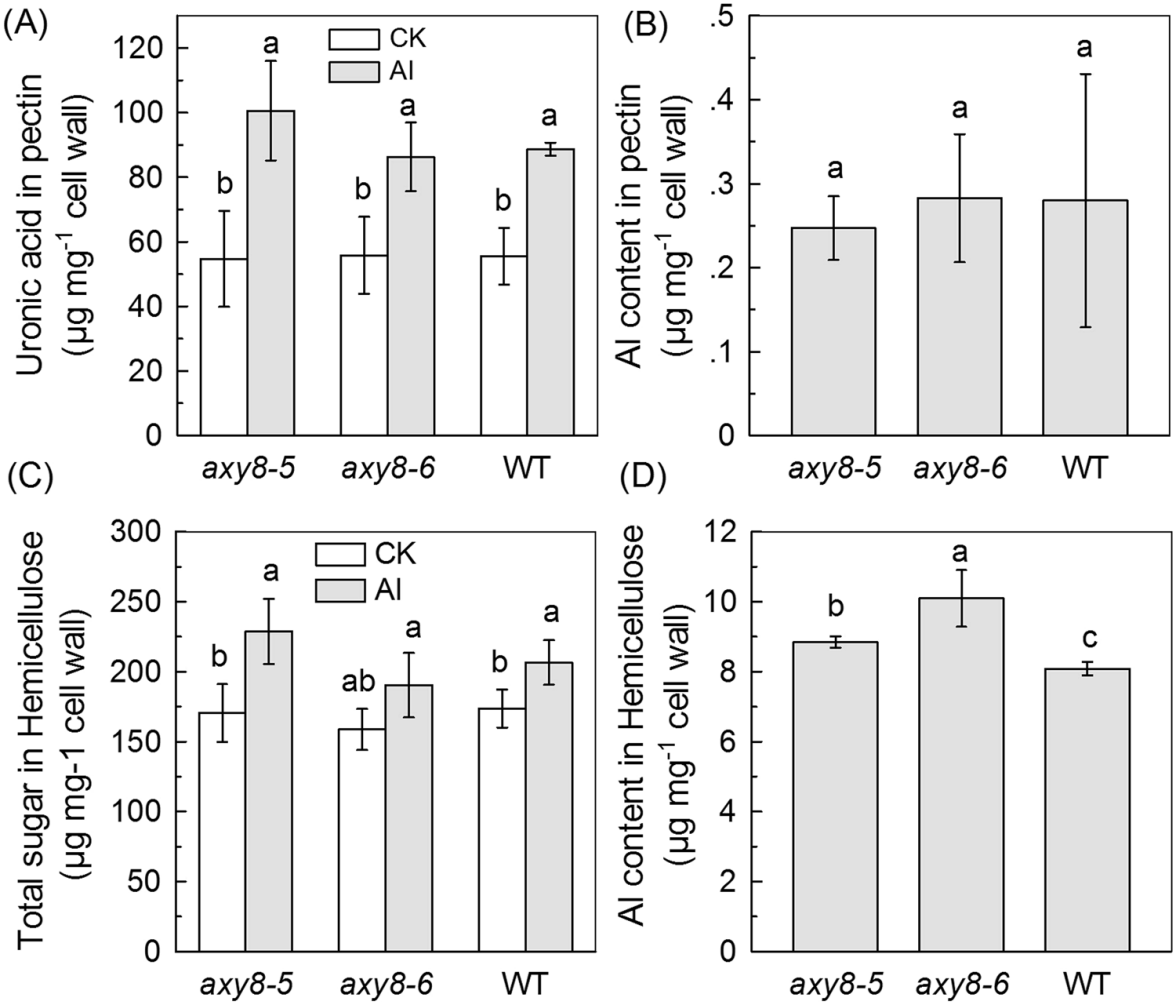
Uronic acid content, total sugar and Al content in pectin and hemicellulose in WT and *axy8* mutants. (**A**) Uronic acid content in the extractable pectin of WT, *axy8-5* and *axy8-6* roots. Seedlings were treated with or without Al for 24 h. (**B**) Al content in the extractable pectin of WT, *axy8-5* and *axy8-6* roots. Cell wall materials from Al treated roots were fractionated into pectin (see Methods for details). (**C**) Total sugar content in extractable hemicellulose of WT, *axy8-5* and *axy8-6* roots. Seedlings were treated with or without Al for 24 h. (**D**) Al content in the extractable hemicellulose of WT, *axy8-5* and *axy8-6* roots. Cell wall materials from Al treated roots were fractionated into hemicelluloses (see Methods for details). Data are means ± SD. n = 4. Different letters show significant differences at P < 0.05 by Student’s *t* test.

Because XyG is the major cell wall Al binding component in Arabidopsis^16^, and because the *axy3* and *axy8* mutants have altered XyG structure, we next measured the Al content of cell wall hemicellulose, and found that the hemicellulose Al content was significantly reduced in the *axy3* mutants (Fig. 6D), but increased in the *axy8* mutants (Fig. 7D). The total hemicellulose sugar content was no different to WT in the *axy3* and*axy8* mutants in the absence of Al treatment (with the exception of the *axy3.2* mutant). However, Al treatments increased the hemicellulose content to a similar level in the WT and in all mutants (Figs. 6C and 7C), suggesting that the different hemicellulose Al contents in the mutants may be due to their altered XyG structure.

Because the pectin cell wall component can also bind Al, due to its carboxyl groups, we next measured both the pectin Al content and the pectin uronic acid content (the latter an indicator for carboxyl group content). We found negligible differences in these between WT, *axy3* and *axy8* mutants (Figs. 6A,B and 7A,B). This observation enables us to exclude the possibility that changes in cell wall pectin contribute significantly to the respectively decreased or increased cell wall Al contents in the *axy3* or *axy8* mutants.

### Determination of intracellular Al levels in the axy3 and axy8 mutants

**Figure 8:**
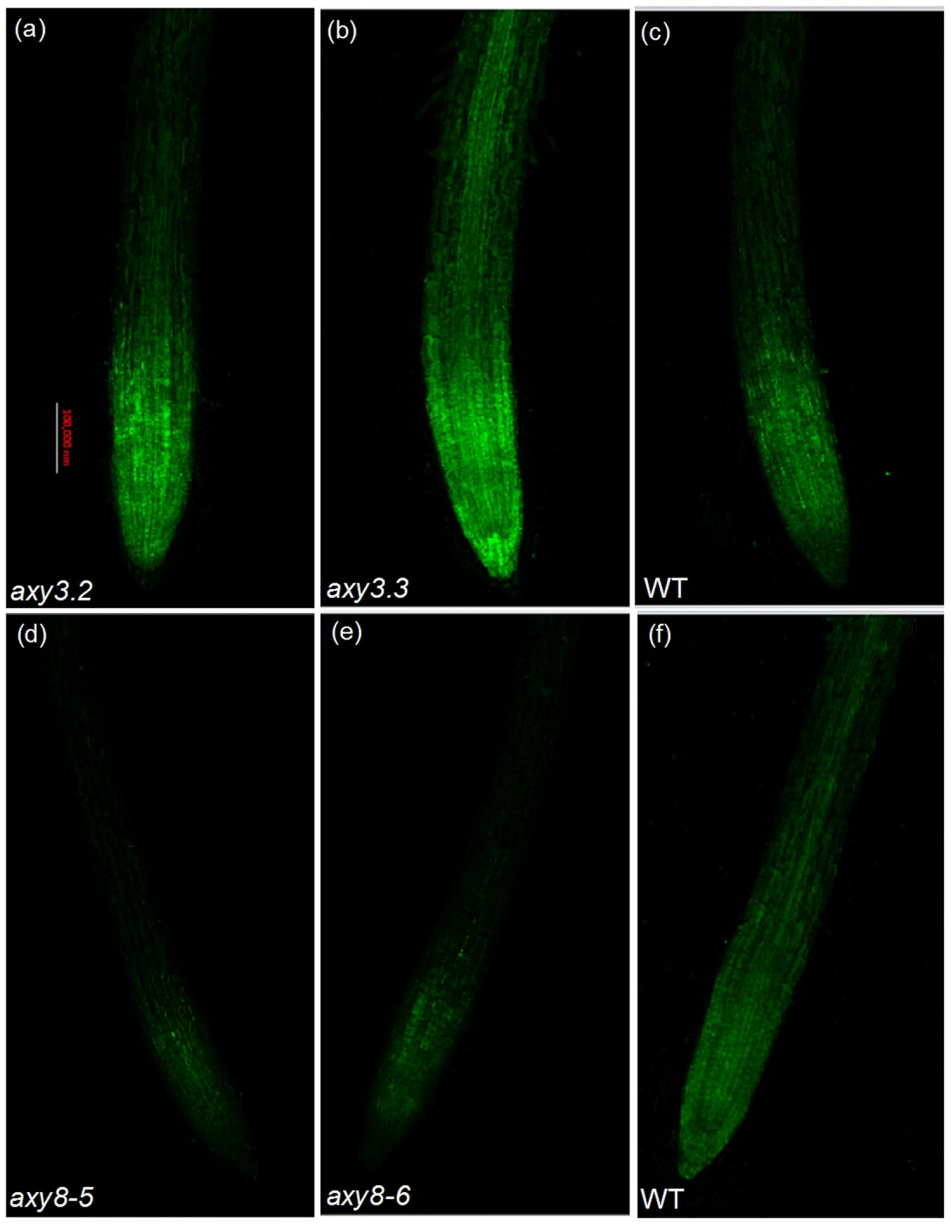
Cellular Al distribution as revealed by morin staining (green). Approximately 1-cm-long seedlings were exposed to 0.5 mM CaCl_2_ solution with 50 μM Al for 24 h. The pH was adjusted to 4.5. Roots were cut between 5 and 10 mm from the apex for morin staining and fluorescence observation. Bar = 0.1 mm.

In a previous report, we showed that if reduced cell wall Al retention is coordinated with increased sequestration of Al into the vacuole, this will cause increased Al resistance^31^. We next used the stain Morin to visualize cytosolic Al content. Binding of cytosolic (but not cell wall-bound or vacuole-compartmentalized) Al to morin elicits detectable green fluorescence^5,32^. The stronger the fluorescence intensity, the higher the cytosolic Al content. We stained root tips with morin following treatment of plants with 50 μM Al for 24 h, and found that *axy3* mutants exhibited stronger than WT Al-dependent green fluorescence, whilst *axy8* mutants exhibited weaker fluorescence (Fig. 8). These differences in fluorescence level reveal large differences in the amounts of Al accumulated in the cytsols of WT, *axy3*, and *axy8* mutants, differences that are negatively correlated with their respective Al sensitivities. In addition, the greater fluorescence exhibited by the *axy3.3* (versus the *axy3.2*) mutant also partially explains its relatively greater Al sensitivity (Fig. 2).

## Discussion

We recently showed that the hemicellulose fraction of root cell walls binds much more Al than does the pectin fraction, and that XyG, the majority component of *Arabidopsis* hemicellulose, contributes to Al binding more than the other hemicellulose components^15,16^. In this present study, we further investigated the relationship between modification of XyG structure and change in XyG Al binding capacity. We showed that reduction in *AXY3* function results in reduced root cell wall Al accumulation and Al adsorption (Figs. 5A and B; Suppl Fig. 1), whilst reduction in *AXY8* function has the opposite effect (Fig. 5C and D). We also showed that *axy3* mutants have increased levels of total root and cytosolic Al content, whilst *axy8* mutants have reduced levels (Figs. 1 and 8). These differences confer the resultant differences in Al sensitivity, with *axy3* mutants being more Al sensitive, and *axy8* more Al resistant. Although no significant difference in hemicellulose (Figs. 6C and 7C) and pectin (Figs. 6A and 7A) contents of WT, *axy3* and *axy8* mutants was detected, there were significant differences in hemicellulose Al contents in plants growth in Al stress conditions (Figs. 6D and 7D). We therefore conclude that XyG fucosylation level, as controlled by the *AXY3* and *AXY8* gene products (AXY3 and AXY8 respectively), affects *Arabidopsis* hemicellulose Al binding capacity.

AXY3 is a member of the *Arabidopsis* carbohydrate active enzyme (CAZy) glycoside hydrolase family 31 (GH31), comprising five proteins^29^. The closest Arabidopsis homolog to *AXY3* is At3g45940, which is not expressed and is likely to be a pseudogene^33^. However, besides the AXY3 tested in this study, the remaining *Arabidopsis* GH31 proteins likely act as α-glucosidases, due to their homology to mammalian and fungal α-glucosidases^34^. In contrast, AXY8 (Fuc95A; At4g34260) is the single member of the CAZy hydrolase family 95 found in Arabidopsis^19^ and acts as a dominant apoplastic XyG: α-Fucosidase. We found that the expression of both of the *Arabidopsis AXY3* and *AXY8* genes is responsive to Al stress (Fig. 4). The down-regulation of *AXY3* and *AXY8* expression by Al (especially that of *AXY3*) suggests the possibility that the AXY3 and AXY8 may differentially modify xyloglucan side chains in the cell wall in response to Al stress, and that this relates to the Al-induced inhibition of cell expansion.

To survive exposure to potentially toxic environmental Al, plants need to be able to avoid direct exposure of vital internal structures and metabolic processes to Al ions^35^. Physiological mechanisms of Al resistance are well known to be achieved either via exclusion of Al from the root symplasm (restriction of Al uptake) or via intracellular tolerance of Al (tolerance of symplastic Al)^36^. Substantial evidence indicates that Al sensitivity is negatively correlated with root Al content in a variety of genotypes^14,37^ or mutants/transgenic lines^16,38^. In our previous study, a T-DNA insertional mutant (*xth31*) was shown to accumulate significantly less Al in its roots, due to its greatly reduced XyG content. In consequence, the *xth31* mutant is strongly Al resistant^16^. However, in a more recent study, we showed that if the total root Al content in different *Arabidopsis* mutants is similar, their Al sensitivities are not only dependent on the cell wall Al content, but also on the relative distribution of Al in the cytosol and the vacuole^31^. It is noteworthy that endocytosis is also proposed to be a process relevant for the uptake of Al into root apex cells^39^. Because both classes of cell wall Al-binding molecules (demethylated pectins and fucosylated XyG) are enriched within trans-Golgi network/early endosomes (TGN/EE), it is possible that Al is internalized via endocytosis of these molecules, especially in the cells of the root apex transition zone where the cells have very high endocytosis activity^39,40^. Here, we found that *axy3* has a higher total root Al content than WT (Figs. 2 and 3), but because less Al was retained in the cell wall (Fig. 5), more Al might enter into root cells. However, the reduced levels of fucosylated XyG in *axy3* may reduce its cell wall Al binding capacity on the one hand and reduce the possible endocytosis within the cell on the other hand, thus more Al may be presented in cytosol and renders *axy3* more Al sensitive. Therefore, plant resistance to Al stress still relies on the proper operation of internal detoxification mechanism to sequestrate Al into vacuole or other metabolic inactive organs or tissues.

Amongst all cell wall matrix components, hemicellulose has been shown to be the major Al binding component^15^. Recently, it was reported that treatment with sodium nitroprusside (SNP, a NO donor) or with auxin can alleviate Cadmium (Cd)-induced inhibition of root elongation. These treatments increase the root cell wall hemicellulose content, causing increased levels of Cd to be retained in root cell wall hemicellulose, and, in turn, causing reduced levels of Cd to be transported to the shoots^41^. In our previous report, we showed that XyG, the key *Arabidopsis* hemicellulose component, is the major Al binding component^16^. In addition, acetylation can protect polysaccharides from enzymatic digestion^42^. Accordingly, we also showed that mutants with reduced *O*-acetyltransferase activity (lower level of *O*-acetylation XyG substitution) accumulate increased levels of Al in their cell walls^31^. These observations suggest that the acetylation of XyG affects its Al binding capacity. In this present study, we further explored the possibility that change of side-chain length via fucosylation affects the Al binding capacity of hemicellulose, by testing mutants with reduced higher or increased fucosylation levels. Our results showed that *axy8* mutants with (increased XyG fucosylation) accumulated more Al whilst *axy3* mutants (reduced XyG fucosylation) accumulated less Al in hemicelluloses (versus WT controls; Fig. 6). These observations demonstrate the importance of differential XyG fucosylation in modulating the Al binding capacity of the root cell wall. The structure of XyG is modified by the length of side chain^43^, which may change its susceptibility to XTH activity^44,45^ and/or its binding to cellulose^46^. Park and Cosgrove^47^ have speculated that the side chains may affect interactions with cellulose and possibly with other components. Nevertheless, how the fucosylated substitutions on XyG side chains affect the amount of Al that XyG can bind still needs to be determined in the future. In conclusion, our study demonstrates that the level of XyG fucosylation is causally and positively correlated with root cell wall Al binding capacity, and that this capacity is crucial to *Arabidopsis* Al resistance.

## Materials and Methods

### Plant Material and Growth Conditions

All wild-type, mutant and transgenic Arabidopsis (*Arabidopsis thaliana*) plants used were in the Col-0 (Columbia ecotype) background. Seeds were vernalized at 4 °C for 2 d. Following surface-sterilization, seeds were germinated on an agar-solidified nutrient medium in Petri dishes. The nutrient solution consisted of the following macronutrients in mM: KNO_3_, 6.0; Ca(NO_3_)_2_, 4.0; MgSO_4_, 1; NH_4_H_2_PO_4_, 0.1, and the following micronutrients in μM: Fe(III)-EDTA, 50; H_3_BO_3_, 12.5; MnSO_4_, 1; CuSO_4_, 0.5; ZnSO_4_, 1; H_2_MoO_4_, 0.1; NiSO_4_, 0.1. The final pH was adjusted to 4.5 with 1 M HCl. Petri dishes were placed into in an environmentally controlled growth chamber or room, positioned vertically. All seedlings were grown at 24 °C, 140 µmol m^−2^ s^−1^ and in a 16/8 h day/night rhythm as previously described^31^.

For hydroponic culture, following 2 weeks growth on the above agar-solidified medium, young plantlets were transplanted to a vermiculite substrate and supplied with nutrient solution for an additional 3 weeks. Seedlings of similar rosette diameters were then transferred to the nutrient solution for a further week, following which the plants were subjected to the following treatments: CK (0.5 mM CaCl_2_, pH 4.5), Al (50 μM Al in the 0.5 mM CaCl_2_, pH 4.5). After 24 h, the roots were excised for RNA extraction. The seedlings were washed three times with deionized water and cut into shoots and roots for Al content analysis, and the fresh weight was also recorded.

For the Al toxicity assay, the above nutrient solution (and also 0.5 mM CaCl_2_ solution) (with 0.8% agar, pH 4.5) was first sterilized and then cooled to about 50 °C. Next, 50 μM Al in the form of AlCl_3_.6H_2_O was added (following sterilization by filtration) thus making an Al-containing agar medium. Agar-solidified nutrient medium-grown seedlings with a root length of about 1 cm were selected and transferred to Petri dishes containing agar-solidified CaCl_2_ (0.5 mM at pH 4.5) medium containing 0 or 50 µM AlCl_3_ for 0, 0.5, 1, 3, 6, 12 and 24 h for a time course experiment, or 0, 5, 10, 25, 50, 100 and 150 μM AlCl_3_ for a 24 h for dose-response experiment. For long-term treatment, the seedlings were transferred to the agar-solidified nutrient medium containing 0 or 50 µM Al for 7 d. Root length measurements were performed using a digital camera connected to a computer. Data were quantified and analyzed by Photoshop 7.0 (Adobe Systems).

### Gene Expression Analysis

Total RNA was isolated using TRIzol (Invitrogen). cDNA was prepared from 1 µg of total RNA using the PrimeScript RT reagent kit (Takara). For real-time RT-PCR analysis, 1 µL of 10-fold-diluted cDNA was used for the quantitative analysis of gene expression performed with SYBR Premix ExTaq (Takara) with the following pairs of gene-specific primers (*AXY3*: forward: 5′- TCCGGAAATGAAGCTAGGAA-3′; reverse: 5′-GCTCCTTCGAGCTAACCTCA-3′, for *AXY8*: forward: 5′-GTCAACCACCTGGAAAGC-3′; reverse: 5′-TCCGACCAAAGACCAAACT-3′ and for *tubulin*: forward: 5′-AAGTTCTGGGAAGTGGTT-3′; reverse: 5′-CTCCCAATGAGTGACAAA-3). Each cDNA sample was run in triplicate. Expression data were normalized with respect to the expression level of the *tubulin* gene.

### Root Cell Wall Extraction and Fractionation

Extraction of root crude cell wall materials and subsequent fractionation of cell wall components was carried out as previously described^18^. Briefly, root samples were ground with a mortar and pestle in liquid nitrogen and then homogenized with 75% ethanol for 20 min in an ice-cold water bath. The sample was then centrifuged at 10,000 g for 10 min and the supernatant was removed. The pellets were homogenized and washed with acetone, methanol: chloroform at a ratio of 1:1, and methanol, respectively, for 20 min each, with each supernatant removed after centrifugation between the washes. The remaining pellet, i.e., the cell wall material, was dried and stored at 4 °C for further use.

Pectin was extracted three times from the above extracted cell wall material with 1 mL hot water at 100 °C for 1 h each and the extracts combined. Next, the hemicellulose fraction was extracted twice with 1 mL 4 M KOH containing 0.02% (w/v) KBH_4_ at room temperature for 12 h.

### Uronic acid and total polysaccharide measurements

Pectin uronic acid content was assayed according to Blumenkrantz and Asboe-Hansen^48^ using galacturonic acid (Sigma) as a standard. Briefly, 200 μL pectin extracts (the total volumn was 3 mL) were incubated with 1 mL 98% H_2_SO_4_ (containing 0.0125 M Na_2_B_4_O_7_·10H_2_O) at 100 °C for 5 min. Following cooling, 20 μL M-hydro-dipheny (0.15%) was added to the solution. The sample was then allowed to stand at room temperature for 20 min before the absorbance at 520 nm was measured spectrophotometrically.

The total polysaccharide contents in the hemicellulose fractions were determined by the phenol sulfuric acid method^16^ and expressed as glucose equivalents. Briefly, 200 μL hemicellulose (HC) extracts (the total volumn was 2 mL) were incubated with 1 mL 98% H_2_SO_4_ and 10 μL 80% phenol at room temperature for 15 min, then incubated at 100 °C for 15 min. Following cooling, the absorbance at 490 nm was measured spectrophotometrically.

### Al Content Measurement

For total Al content determination, roots were harvested and digested with HNO_3_:HClO_4_ (4:1, v/v). For cell wall Al content determination, Al was extracted with 2 N HCl for at least 24 h with occasional shaking. Al concentration in the extracts was determined by inductively coupled plasma-atomic emission spectrometry (ICP-AES; IRIS/AP optical emission spectrometer).

### Adsorption Kinetics

In order to determine the ability of different cell wall components to adsorb Al, a total of 5 mg cell wall materials was placed in a 2-mL column equipped with a filter at the bottom as previously described^18^. The adsorption solution consisted of 20 µM AlCl_3_ in 0.5 mM CaCl_2_ at pH 4.5. The solution was passed through the bed of cell wall driven by a peristaltic pump at 12 mL h^−1^. The eluates were collected in 4-mL aliquots, which were assayed for Al spectrophotometrically with pyrocatechol violet according to Kerven *et al*.^49^.

### MALDI–TOF MS Analysis of Xyloglucan Oligosaccharides

Alcohol insoluble residues (AIRs) were generated from roots of Col-0 in the presence or absence of 50 μM Al for 24 h, and then de-starched with α-amylase (*Bacillus* sp.). The xyloglucan-enriched KOH-soluble fraction was prepared by treating 50 mg of de-starched AIRs in 4 M KOH solution and lyophilized after neutralization and dialysis. Then, 0.5 mg of the AIRs or the KOH fraction were incubated in 100 μL of 50 mM ammonium formate (pH 5.0), with one unit of xyloglucanase (E-XEGP, Megazyme, Wicklow, Ireland) for 18 h at 37 °C. The supernatants were recovered, and 1 μL of aqueous sample plus 10 ng xylopentaose (Megazyme, Wicklow, Ireland) was spotted with an equal volume of matrix solution (10 mg mL^−1^ 2,5-dihydroxbenzoic acid). Following drying onto the MALDI target plate, spectra were analyzed on a Bruker Autoflex MALDI–TOF MS instrument (Bruker, www.bruker.com/) in the positive reflection mode with an acceleration voltage of 20 kV. The relative height of each generated oligosaccharide ion peak was counted to determine their relative abundance as described in Zhang *et al*.^50^.

### Statistical analysis

Each experiment was repeated at least three times. Data were analyzed by one-way ANOVA procedure and the means were compared by Duncan’s multiple range test. Different letters on the histograms represent statistically different values at the P < 0.05 level.

## Electronic supplementary material

Supplemental figure
