# Supplementary material for: Xyloglucan Fucosylation Modulates Arabidopsis Cell Wall Hemicellulose Aluminium binding Capacity
Source: Sci Rep. 2018 Jan 11;8:428. doi: 10.1038/s41598-017-18711-1 (PMC5765015; doi:10.1038/s41598-017-18711-1)

Xyloglucan Fucosylation Modulates *Arabidopsis* Cell Wall Hemicellulose Aluminium binding Capacity

Jiang-Xue Wan[1], Xiao-Fang Zhu[2], Lin-Yu Liu[1], Yu-Qi Wang[1], Bao-Cai Zhang[3], Gui-Xin Li[4], Yi-Hua Zhou[3] and Shao-Jian Zheng[1,] *

Supplemental Figure 1. Quantitative RT-PCR analysis of *AXY3* (A) and *AXY8* (B) expression in *Arabidopsis* whole roots. Expression levels were compared with the expression level of *tubulin* that was assigned as expression level of 1. Data are means ± SD. n=4. Different letters show significant differences at $P < 0.05$ by Student's *t* test.

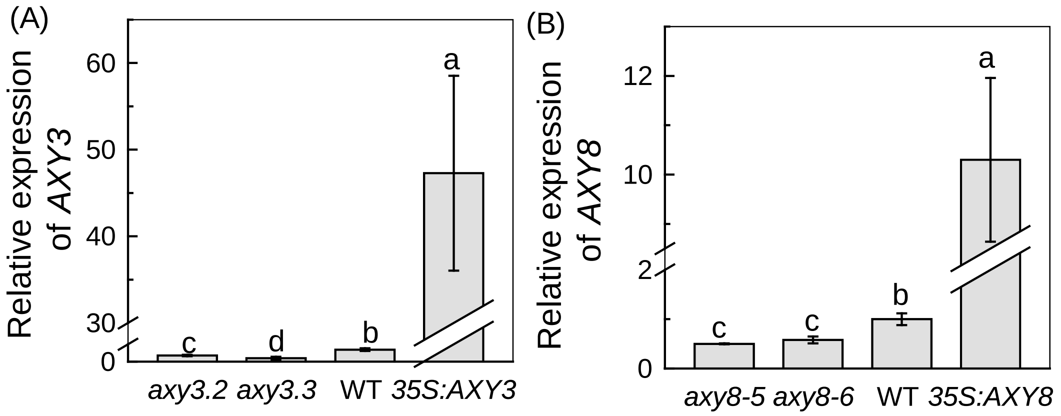

Supplement: Supplementary file 1 — Supplemental figure [file 41598_2017_18711_MOESM1_ESM.pdf]
